# Egyptian H5N1 Influenza Viruses—Cause for Concern?

**DOI:** 10.1371/journal.ppat.1002932

**Published:** 2012-11-15

**Authors:** Gabriele Neumann, Catherine A. Macken, Alexander I. Karasin, Ron A. M. Fouchier, Yoshihiro Kawaoka

**Affiliations:** 1 Department of Pathobiological Sciences, University of Wisconsin-Madison, Madison, Wisconsin, United States of America; 2 Theoretical Division, Los Alamos National Laboratory, Los Alamos, New Mexico, United States of America; 3 Department of Virology, Erasmus Medical Center, Rotterdam, The Netherlands; 4 ERATO Infection-Induced Host Responses Project, Saitama, Japan; 5 Department of Special Pathogens, International Research Center for Infectious Diseases, Institute of Medical Science, University of Tokyo, Tokyo, Japan; 6 Department of Microbiology and Infectious Diseases, Kobe University, Hyogo, Japan; 7 Division of Virology, Department of Microbiology and Immunology, Institute of Medical Science, University of Tokyo, Tokyo, Japan; The Fox Chase Cancer Center, United States of America

Highly pathogenic avian H5N1 influenza viruses are now enzootic in parts of Southeast Asia and the Middle East. Occasionally, these viruses transmit to humans and cause severe respiratory disease and fatalities. Currently, these viruses are not efficiently transmitted from person to person, although limited human-to-human transmission may have occurred [1]–[4]. A major determinant of influenza virus host range is the viral hemagglutinin (HA) protein: avian virus HA binds preferentially to sialic acid linked to the penultimate galactose residue by an α2,3-linkage (Siaα2,3Gal) [5]–[7], as found for sialic acid–containing receptors of the epithelial cells in duck intestine [8], the site of avian influenza virus replication. By contrast, human virus HA has higher affinity for Siaα2,6Gal [5]–[7], the main sialyloligosaccharide on the epithelial cells of the human upper respiratory tract [9], [10].

Recently, Herfst et al. [11] and Imai et al. [12] identified H5 HA-possessing viruses that transmit via respiratory droplets among ferrets, an established animal model for influenza virus transmission studies. The H5N1 transmissible virus identified by Herfst et al. [11] possesses three mutations that were intentionally introduced (PB2-627K, which confers efficient replication in mammals [13], and HA-Q222L/G224S (H5 numbering), which confer human-type receptor-binding specificity [14], [15]). The “Herfst virus” also possessed two mutations that emerged during virus passages in ferrets. One of these, HA-T156A, results in the loss of a glycosylation site on the head of the HA; the other, HA-H103Y, localizes to the HA trimer interface. The transmissible virus identified by Imai et al. [12] possesses a mutant HA gene of an avian H5N1 virus and the remaining seven viral genes of a prototypic pandemic 2009 (H1N1) virus. Random mutagenesis of the HA globular head identified two mutations in HA (HA-N220K and HA-Q222L; note that the latter is identical to one of the mutations identified in the “Herfst virus”) that conferred human-type receptor-binding specificity. Virus passages in ferrets resulted in the selection of two additional mutations in HA. One of these, HA-N154D, resulted in the loss of the same glycosylation site as HA-T156A in the “Herfst virus”; the other, T314I, affected HA stability [12]).

Although the Herfst and Imai studies used different experimental strategies and tested viruses of HA/H5 clade 2 or 1, respectively, the results were remarkably similar: the transmissible mutant H5 viruses bound to human-type receptors, lost the glycosylation site at HA-154–156, and acquired an additional mutation in HA that likely increased the protein's stability. Moreover, both studies, and findings by others [16]–[18], suggest that a shift towards human-type receptor-binding specificity may be necessary, but not sufficient, for H5N1 virus transmissibility in mammals.

The loss of a glycosylation site at HA-154–156, using two different mutations, is particularly notable. Amino acids 154–156 of many H5 HAs encode an N-glycosylation site (N-X(except P)-S/T), which is located near the receptor-binding pocket. Loss of this glycosylation site enhances H5N1 virus binding to Siaα2,6Gal (conferred by the Q226L/G228 mutations [19]) and is critical for H5N1 virus transmissibility in guinea pigs [20]. In the Herfst and Imai studies, loss of this glycosylation site occurred during the first virus passages in ferrets, suggesting that this trait is essential for H5 virus transmissibility in ferrets.

Since lack of the HA154–156 glycosylation site appears to be critical for H5 virus transmission in mammals, we inspected avian H5N1 viruses for this feature. A phylogenetic tree of publicly available H5 HA sequences showed that a substantial number of these viruses, distributed across time and geography, lack this glycosylation site. Closer inspection of 2009–2011 H5N1 viruses from Vietnam, Indonesia, and Egypt (i.e., countries with appreciable numbers of human H5N1 infections) revealed that ∼25% of Vietnamese, 0% of Indonesian, but >70% of Egyptian isolates lack the HA154–156 glycosylation site. The H5N1 viruses currently circulating in Egypt are descendants of the so-called Qinghai Lake viruses that killed wild birds (which typically do not succumb to influenza virus infections) at Qinghai Lake, China, in 2005 [21]–[23], and have now spread through Europe to the Middle East and Africa.

Next, we looked for a correlation between the HA154–156 glycosylation site and recent human H5N1 virus infections. Human H5N1 infections in Vietnam and Indonesia from 2009 to 2011 were mostly (Vietnam) or exclusively (Indonesia) caused by viruses with the HA154–156 glycosylation site. However, all 46 H5N1 viruses isolated in 2009–2011 from infected individuals in Egypt lacked the HA154–156 glycosylation site, while 28% of H5N1 viruses circulating in avian species in Egypt in 2009–2011 possessed this site (Table 1). Phylogenetic analysis further suggested that mutations resulting in loss of the glycosylation site occurred in birds and that these variants subsequently transmitted to humans. Although speculative at this point, this finding might suggest that avian H5N1 viruses lacking the HA154–156 glycosylation site transmit to humans more readily than those that possess the glycosylation site, at least in the genetic background of Egyptian H5N1 viruses.

**Table 1:** HA154–156 glycosylation site in Egyptian H5N1 influenza viruses.

Year of Isolation	Avian Isolates					Human Isolates				
	Total Number	Glycosylation Site +		Glycosylation Site −		Total Number	Glycosylation Site +		Glycosylation Site −	
		Number	%	Number	%		Number	%	Number	%
2009	92	64	70	28	30	34	34	100	0	0
2010	132	77	58	55	42	12	12	100	0	0
2011	82	78	95	4	5	0	0	0	0	0
2009–2011	306	219	72	87	28	46	46	100	0	0

The H5N1 sequences evaluated were obtained from the Global Initiative on Sharing Avian Influenza Data (GISAID; http://platform.gisaid.org/epi3/frontend).

In addition to mutations in HA, other amino acid changes may be critical to confer transmissibility in humans. One such mutation may be the glutamic acid-to-lysine mutation at position 627 of the PB2 polymerase protein (PB2-627K), which confers efficient replication in mammals [13] and is a recognized host determinant of influenza viruses [24]. Herfst introduced this mutation into their virus; the “Imai virus” possesses the 2009 pandemic PB2 gene, in which a basic amino acid at position 591 compensates for the lack of PB2-627K [25], [26]. The mammalian-adapting PB2-627K mutation also emerged in the Qinghai Lake viruses [21]–[23] and has been maintained in viruses of this lineage to this day, with the exception of a few revertants. Most Egyptian H5N1 viruses, which descend from the Qinghai Lake viruses, thus possess two mutations that may facilitate transmissibility in mammals: a mutation in HA (resulting in the lack of a glycosylation site) that is critical for H5N1 virus transmissibility in ferrets and guinea pigs, and a mutation in PB2 that confers efficient replication in mammals. In addition, some Egyptian H5N1 viruses have acquired increased affinity for human-type receptors [27].

The data presented here are invaluable for monitoring circulating viruses for variants with increased potential to acquire transmissibility to mammals. Our database searches identified two H5N1 viruses that encode HA-220K and have lost the HA154–156 glycosylation site (A/muscovy duck/Vietnam/NCVD-11/2007; A/duck/Egypt/10185SS/2010), indicating that only two additional mutations are needed to create variants with the “transmissibility features” identified in the Kawaoka study. Because the outbreak of H5N1 viruses in Egypt is extensive, Egyptian H5N1 viruses may, therefore, present a far greater pandemic risk than H5N1 viruses circulating in other countries.
